# Aliphatic Polybenzimidazoles: Synthesis, Characterization and High-Temperature Shape-Memory Performance

**DOI:** 10.3390/polym15061399

**Published:** 2023-03-11

**Authors:** Bato Ch. Kholkhoev, Zakhar A. Matveev, Kseniia N. Bardakova, Peter S. Timashev, Vitaliy F. Burdukovskii

**Affiliations:** 1 Baikal Institute of Nature Management, Siberian Branch of the Russian Academy of Sciences, 670047 Ulan-Ude, Russia; 2 Research Center “Crystallography and Photonics”, Russian Academy of Sciences, 108840 Moscow, Russia; 3 Institute for Regenerative Medicine, Sechenov University, 119991 Moscow, Russia; 4 Semenov Institute of Chemical Physics, Russian Academy of Sciences, 119991 Moscow, Russia; 5 Chemical Department, Lomonosov Moscow State University, 119991 Moscow, Russia

**Keywords:** polybenzimidazoles, high-temperature shape-memory polymers, mechanical properties, thermally stable polymers, physical crosslinking, hydrogen bonding

## Abstract

A series of aliphatic polybenzimidazoles (PBIs) with methylene groups of varying length were synthesized by the high-temperature polycondensation of 3,3′-diaminobenzidine (DAB) and the corresponding aliphatic dicarboxylic acid in Eaton’s reagent. The influence of the length of the methylene chain on PBIs’ properties was investigated by solution viscometry, thermogravimetric analysis, mechanical testing and dynamic mechanical analysis. All PBIs exhibited high mechanical strength (up to 129.3 ± 7.1 MPa), glass transition temperature (≥200 °C) and thermal decomposition temperature (≥460 °C). Moreover, all of the synthesized aliphatic PBIs possess a shape-memory effect, which is a result of the presence of soft aliphatic segments and rigid *bis*-benzimidazole groups in the macromolecules, as well as strong intermolecular hydrogen bonds that serve as non-covalent crosslinks. Among the studied polymers, the PBI based on DAB and dodecanedioic acid has high adequate mechanical and thermal properties and demonstrates the highest shape-fixity ratio and shape-recovery ratio of 99.6% and 95.6%, respectively. Because of these properties, aliphatic PBIs have great potential to be used as high-temperature materials for application in different high-tech fields, including the aerospace industry and structural component industries.

## 1. Introduction

Polybenzimidazoles (PBIs) are a class of high-performance polymers that, due to their chain stiffness and strong intermolecular hydrogen bonds, are characterized by excellent heat resistance, wear resistance, mechanical strength, chemical stability, radiation resistance and dielectric properties [1,2,3,4,5,6]. Due to these properties, PBIs appear to be attractive materials for use in various fields, such as aerospace, the petrochemical industry, electronic devices, gas separation, fuel cells, etc. [1,2,3,7,8,9,10,11,12,13,14,15,16,17,18].

In order to expand the possibilities of the practical application of PBIs, it is necessary to impart additional functional properties to materials based on them, such as the high-temperature shape-memory effect. High-temperature shape-memory polymers (SMPs) have great potential for practical applications in the development of deployable space structures, smart jet propulsion systems, engine controls and high-temperature actuators [19,20,21,22,23,24]. SMPs contain rigid segments that can be formed by covalent crosslinking or hydrogen bonding, as well as crystalline, liquid-crystalline or amorphous soft block domains. However, traditional PBIs such as poly-2,2′-*m*-phenylene-5,5′-dibenzimidazole have highly rigid backbones and do not contain any flexible groups, so they do not possess a shape-memory effect. Previously, we showed that high-temperature SMPs could be obtained from semi-interpenetrating polymer networks based on fully aromatic poly-2,2′-*p*-oxydiphenylene-5,5′-dibenzimidazole (OPBI) [25]. In [26], we showed that a PBI with flexible aliphatic octamethylene fragments possesses good shape-memory ability, in contrast to poly (imide-*co*-benzimidazole) [27] or OPBI [26].

It is known that the properties of aliphatic PBIs largely depend on the number of methylene fragments in the dicarboxylic acid used for the synthesis [28,29,30,31,32,33]. In addition, the reactivity of dicarboxylic acids significantly depends on the length of the methylene chain, which can affect the molecular weight characteristics and, as a result, the properties of the obtained polymers. As shown previously, the polycondensation of 3,3′-diaminobenzidine (DAB) and a number of aliphatic dicarboxylic acids in polyphosphoric acid leads to high-molecular-weight PBIs, and their molecular weight increases with the lengthening of the aliphatic chain of the dicarboxylic acid [31]. Moreover, a number of authors noted that the introduction of aliphatic fragments into PBI macromolecules leads to a decrease in chain rigidity compared with fully aromatic PBI, while the thermal stability decreases slightly [29,31], and the oxidative stability increases significantly [34]. As a result of changing the length of aliphatic fragments, the ratio of rigid and flexible segments changes, which can significantly affect the ability of the material to fix a temporary shape and recover its original shape. In a number of reports [35,36,37], it was shown that by varying the ratio of hard and soft segments, as well as the concentration of crosslinking nodes, the shape-fixity ratio (R_f_) and shape-recovery ratio (R_r_) can vary in a wide range.

Based on the above, in view of developing applications of aliphatic PBIs as a new class of high-temperature SMPs, the systematic study of the structure–property relationships of PBIs derived from different aliphatic dicarboxylic acids seems to be relevant. In this work, we synthesized a number of aliphatic PBIs using aliphatic dicarboxylic acids with an increasing number of –CH_2_– groups (4, 7, 10 and 14) as monomers. The resulting PBIs have been comprehensively characterized by various physicochemical methods, and the effect of the aliphatic chain length on the thermal, mechanical and thermomechanical characteristics, as well as the shape-memory performance of the materials based on them, has been established.

## 2. Experimental Section

### 2.1. Materials

DAB (≥97%), adipic acid (99%), azelaic acid (98%), dodecanedioic acid (99%), hexadecanedioic acid (96%), methanesulfonic acid (≥99%) and P_2_O_5_ (99%) were obtained from Sigma-Aldrich (St. Louis, MO, USA) and used as received. All solvents were received from local suppliers and purified by common methods. Eaton’s reagent (ER) was prepared according to a previously described procedure [26].

### 2.2. PBI Synthesis

PBIs were synthesized by the polycondensation of DAB and dicarboxylic acid (Table 1) using ER as a reaction medium. A three-necked flask was charged with ER, DAB and an equimolar quantity of the dicarboxylic acid at room temperature. The monomer concentration is dependent on the dicarboxylic acid used (Table 1). The temperature of the reaction mixture was slowly raised to 120 °C and maintained for a certain duration, as given in Table 1. After the required time, the formed viscous solution was poured into the stirred aqueous ammonia solution (0.01 N, pH = 10–11). The precipitated polymer was washed with water and kept overnight in aqueous ammonia to extract residual acids from the polymer. The resulting polymer was filtered and washed with water until the filtrate was neutral. Finally, the obtained PBI was dried in vacuo at 150 °C for 24 h. The reduced viscosities (η_red_) of the obtained PBIs are presented in Table 1. PBI film materials were obtained by the solution-casting method using a 3.5% (*w*/*v*) polymer solution in FA. After two days, the formed film was peeled off from the Petri dish, immersed in an aqueous ammonia solution (0.01 N, pH = 10–11) for 24 h in order to remove the residual acid and then kept in water for 2 days. The films were dried in a vacuum oven at 150 °C for 2 days.

### 2.3. Analytical Methods

The reduced viscosity (η_red_) was measured with an Ostwald viscometer at 20 °C in a constant-temperature water bath. FA was used as a solvent, and the polymer concentration was 0.5 g/dL. ATR FTIR spectra were obtained with an FT-IR Spectrum Two spectrometer (PerkinElmer Inc., Waltham, MA, USA) in the wavenumber range of 4000–400 cm^–1^. Wide-angle X-ray diffraction (WAXD) patterns and intensity traces around the azimuth were recorded with a D2 Phaser (Bruker, Billerica, MA, USA) diffractometer.

The materials’ mechanical properties were studied using an Instron 3367 (Norwood, MA, USA) testing machine at a stretching rate of 1 mm/min. Their thermal properties were evaluated by TGA in an argon atmosphere at a heating rate of 10 °C/min using an STA 449 F3 (Netzsch, Selb, Germany) thermal analyzer. The thermomechanical behavior of PBIs was investigated using a DMA242C (Netzsch, Selb, Germany) analyzer at a frequency of 1 Hz, a dynamic force of 1 N and a heating rate of 10 °C/min.

The shape-memory properties of the samples were studied with the same DMA analyzer. Four consecutive cycles of the shape-memory test were carried out for each sample. The size of the samples was 30 mm × 5 mm × 0.1 mm. The shape-memory test consisted of the following stages: (a) heating the sample to the programmed temperature (T_prog_ = T_g_ + 30); (b) applying a force for the elongation of the sample; (c) lowering the temperature to 100 °C to fix the temporary shape; (d) removing the force; (e) reheating the sample to the recovery temperature (T_rec_ = T_g_ + 30). The cycle was then repeated by using the same regime.

## 3. Results and Discussion

### 3.1. Synthesis and Characterization of PBIs

**Figure 1:**
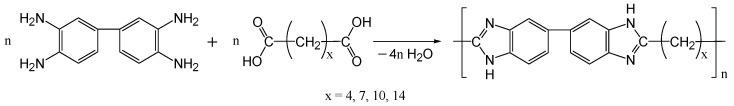
Scheme of the synthesis of aliphatic PBIs.

Aliphatic PBIs were synthesized by the high-temperature polycondensation of DAB and four aliphatic dicarboxylic acids according to the scheme presented in Figure 1. Previously, it was shown [31] that the length of the methylene chain has a significant effect on both the optimal conditions for polycondensation in polyphosphoric acid and the molecular weight of the resulting polymers. Generally, polycondensation in ER instead of polyphosphoric acid produces higher-viscosity PBIs capable of forming mechanically strong film materials [26,30,32]. Taking into account the objectives of this work, this approach seemed to be the most appropriate for obtaining high-performance shape-memory aliphatic PBIs.

**Figure 2:**
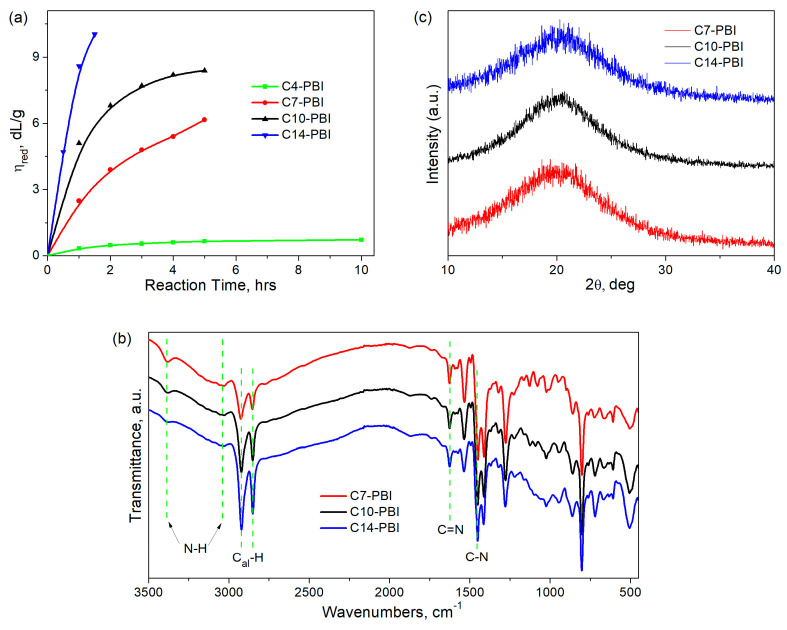
Effect of reaction time on reduced viscosity of PBIs, obtained by polycondensation in ER (**a**). FTIR spectra (**b**) and WAXD patterns (**c**) of PBIs. Dotted lines display the characteristic absorption bands of aliphatic PBIs.

Figure 2a shows the effect of the polycondensation time in ER at 120 °C on the reduced viscosity of aliphatic PBIs. In all cases, polycondensation proceeded in a homogeneous solution and gave quantitative yields of aliphatic PBIs with reduced viscosities up to 10.03 dL/g (at 20 °C, 0.5 g/dL in FA). It should be noted that, in the case of adipic acid, the polycondensation product (C4-PBI) precipitates in water as a powder and has the lowest viscosity (0.66–0.72 dL/g, Table 1), even with an increase in the duration of the synthesis up to 10 h. Y. Iwakura et al. also noted similar observations of the lowered viscosity of C4-PBI obtained by polycondensation in polyphosphoric acid [28]. This may be explained by the fact that adipic acid can participate in some side reactions with the formation of cyclic anhydride or cyclopentanone. Due to its low molecular weight, C4-PBI does not form stable films, so its further study was not appropriate.

Increasing the number of –CH_2_– groups in dicarboxylic acid monomers up to 7 and 10 results in PBIs with much higher viscosities (Table 1, Figure 2a), while in the case of dodecanedioic acid, a higher-molecular-weight polymer is formed. During the polycondensation of DAB and hexadecanedioic acid at a monomer concentration of 0.2 mol/L, intense gelation occurred with 30 min of synthesis at 120 °C, and the resulting C14-PBI was insoluble even in concentrated sulfuric acid. Only a 1.5-fold decrease in the concentration of the initial monomers and a reduction in the synthesis duration to 1.5 h made it possible to obtain a high-molecular-weight polymer (η_red_ = 10.03 dL/g) readily soluble in sulfuric and formic acids. Our results are in good agreement with the data obtained by other authors [30,31], who noted an increase in the reactivity of aliphatic dicarboxylic acids with an increase in the number of methylene fragments. It should also be noted that high-molecular-weight aliphatic PBIs are formed at a lower synthesis temperature compared to aromatic ones (for example, OPBI), which require synthesis temperatures of 140–150 °C to be obtained [30,38].

The solubility test of aliphatic PBIs (Table S1, Supplementary Data) shows that they were insoluble in NMP, DMAc and DMF and only highly soluble in concentrated sulfuric and formic acids. C7-PBI and C10-PBI were soluble in DMSO at higher temperatures (~100 °C). However, upon cooling, they precipitated again from the solution. For this reason, it was not possible to register the NMR spectra of aliphatic PBIs.

In the FTIR spectra of the aliphatic PBIs (Figure 2b), characteristic absorption bands in the region at ~1630–1450 cm^−1^ corresponding to a benzimidazole ring were observed. The visible peaks at ~2850 and 2950 cm^−1^ could be attributed to C–H vibrations in methylene groups. It should be noted that the intensity of these bands significantly increases with the increase in the number of methylene groups in macromolecular chains. The broad band at ~3050 cm^−1^ could be attributed to the hydrogen-bonded benzimidazole rings due to N–H⋯N interactions, while the peak at ~3400 cm^−1^ could be ascribed to free N–H groups. Since the band corresponding to hydrogen-bonded N–H groups is present in the spectra of all studied aliphatic PBIs, it can be concluded that an increase in PBI chain flexibility due to an increase in the number of methylene groups does not hinder the formation of strong intermolecular hydrogen bonds. Such strong intermolecular hydrogen bonds could serve as non-covalent crosslinking points in the polymer network, which are important in terms of the shape-memory effect [26,27].

Since intermolecular interactions can strongly affect the chain packing of macromolecules, WAXD analysis was further carried out. As can be seen in Figure 2c, all aliphatic PBIs demonstrate only a broad halo, indicating their amorphous nature. The d-spacing values calculated from Wulff–Bragg’s equation using 2θ values corresponding to the halo maxima vary in a narrow range (~4.4–4.6 Å) and are virtually independent of the length of the methylene fragment. On the one hand, the incorporation of aliphatic groups in PBI macromolecules results in an increase in d-spacing compared with fully aromatic PBIs (~3.5–4.2 Å [31,34]). On the other hand, the chain packing of aliphatic PBIs is still tighter than that of other high-performance polymers (≥5.0 Å [39,40,41]), which is a result of intermolecular hydrogen bonding between benzimidazole rings.

### 3.2. Thermal and Mechanical Properties

**Figure 3:**
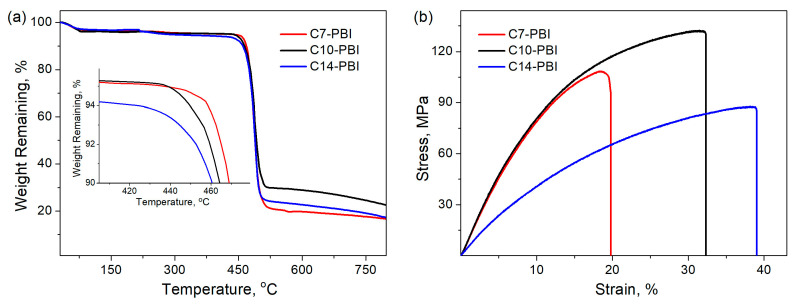
TGA curves (**a**) and stress–strain curves (**b**) of PBIs.

Figure 3a presents the TGA curves of aliphatic PBIs, and the results are summarized in Table 2. All studied PBIs demonstrate ~5% weight loss at ~100 °C, which could be attributed to the elimination of absorbed water. In the temperature range of ~100–450 °C, no significant weight loss is observed, whereas a further increase in temperature results in a fast one-step decomposition, which results in a char yield of 17–23%. Moreover, with an increase in the number of methylene units in the polymer chain, only a slight decrease in the temperature of 10% weight loss (T_10%_) is observed (Table 2). These results are in good agreement with the previous works of several groups [28,29,31], who reported that the variation in the number of –CH_2_– groups has little influence on the thermal stability of aliphatic PBIs. Thus, TGA measurements indicate that aliphatic PBIs have high thermal stability for use in high-performance applications.

The tensile properties of aliphatic PBIs were evaluated by mechanical testing, and the results are presented in Table 2 and Figure 3b. As can be seen, all studied aliphatic PBIs have excellent mechanical durability and demonstrate similar values of tensile strength to conventional PBIs [31,34] but a noticeably higher elongation at break, which is due to the increased flexibility of the backbone with aliphatic units. Moreover, an almost linear increase in the elongation at break is observed with an increase in the number of methylene groups. An interesting observation is that, in the case of tensile strength, we cannot see such linear regularity. Among the investigated polymers, C10-PBI has the highest tensile strength of 129.3 ± 7.1 MPa, while for C7-PBI and C14-PBI, the tensile strength does not exceed 100 MPa. The tensile strength for C10-PBI is reported to be 71.7 MPa for a sample prepared in polyphosphoric acid [31]. It seems that C10-PBI from the present research surpasses the previously reported one, as well as C7-PBI, in terms of its mechanical properties due to higher molecular weight. Several authors have previously noted that PBI films can show improved mechanical properties (higher stress at break) with increasing molecular weight [1,42]. On the other hand, in the case of C14-PBI, a more important role is played by the increased flexibility of the polymer backbone. R. S. Bhavsar et al. presented similar results, showing a decrease in tensile strength with an increase in the number of methylene groups in PBIs [31].

### 3.3. Dynamic Mechanical Properties of PBIs

**Figure 4:**
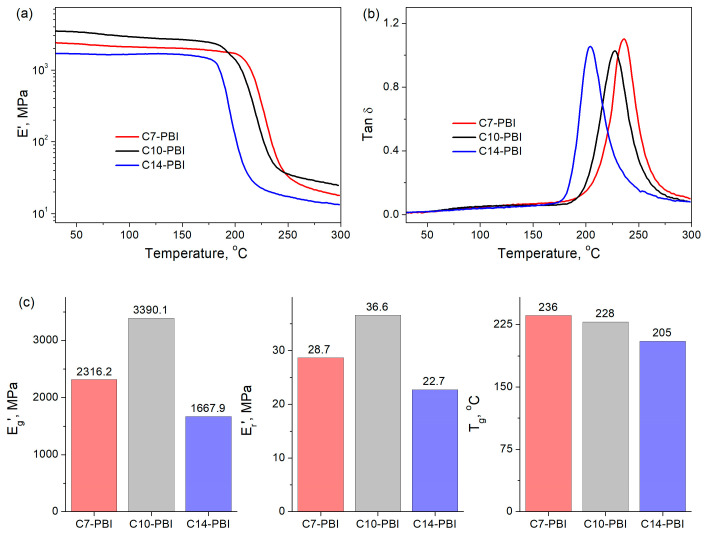
Temperature dependence of storage modulus E’ (**a**) and Tan δ (**b**) of PBIs according to DMA. Summary of E_g_’, E_r_’ and T_g_ of PBIs (**c**).

In the next step of this work, DMA was carried out to evaluate the thermomechanical behavior of aliphatic PBIs (Figure 4). As can be seen in Figure 4a, in all cases, the storage modulus (E’) remains almost unchanged with the increase in temperature in a glassy state, and a sharp drop of about two orders of magnitude is observed around the glass transition temperature (T_g_). This sharp transition from a glassy state to a rubbery one results in a narrow peak in the temperature dependence of Tan δ (Figure 4b). DMA results demonstrate that aliphatic PBIs show facile phase switching, which is important for the appearance of the shape-memory effect [35]. Similar results were observed in our previous work on shape-memory PBI-based materials [25,26].

The E’ values at 50 °C (glassy state, E_g_’) and T_g_ + 20 °C (rubbery state, E_r_’) for C10-PBI are the highest among the studied aliphatic PBIs (Figure 4a,c). These results are in good agreement with a previously conducted mechanical test and are related to the increased molecular weight of C10-PBI in comparison with that of C7-PBI and its smaller number of methylene groups compared to C14-PBI. It should be noted that the E_r_’ values of the presented PBIs are higher than for other shape-memory polyimides [27,43,44]. This could be due to the strong hydrogen bonding in the PBI cases.

T_g_ values were determined from the temperature dependence of Tan δ (Figure 4b,c). As can be seen, all aliphatic PBIs have significantly lower T_g_ in comparison to fully aromatic ones [5,31,42,45], which is attributed to the increased flexibility of the macromolecules containing aliphatic groups. Moreover, a general reduction in T_g_ from C7-PBI to C14-PBI was observed, which could be due to the decrease in macromolecular rigidity with an increase in the number of methylene units.

### 3.4. Shape-Memory Performance of PBIs

The shape-memory performance of aliphatic PBIs was evaluated by the DMA test in tensile mode, and T_prog_ and T_rec_ were set at T_g_ + 30 °C. R_f_ and R_r_ are two quantitative parameters to evaluate the shape-memory effect of polymeric materials. R_f_ represents the ability of SMPs to maintain their acquired shapes as a result of their soft segments and can be calculated using Equation (1), while R_r_ represents the ability of SMPs to recover their permanent shapes and can be calculated by Equation (2).

R_f_ (*n*) = [ε_2_(*n*)/ε_1_(*n*)] × 100%; (1)

R_r_ (n) = [(ε_2_(*n*) − ε_re_(*n*))/(ε_2_(*n*) − ε_re_(*n* − 1))] × 100%; (2)

where ε_2_(*n*) is the strain in the fixed temporary shape after removing the force, ε_1_(*n*) is the strain after stretching the sample under a holding force, and *n* is the cycle number; ε_re_ is the residual strain of the sample at the end of the shape-memory cycle; and ε_re_(*n* − 1) is the ε_re_ of the previous cycle (for the first cycle ε_re_(*n* − 1) = ε_0_).

**Figure 5:**
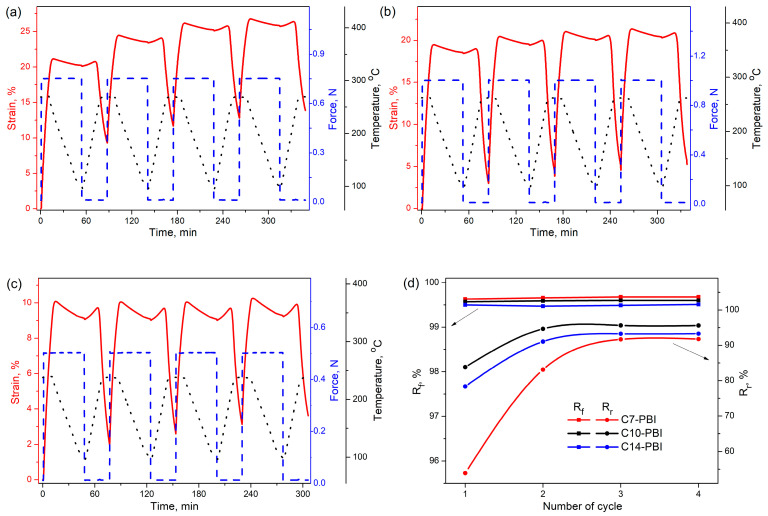
Representative shape-memory cycles of C7-PBI (**a**), C10-PBI (**b**) and C14-PBI (**c**). Cycle vs. shape-memory properties of PBIs (**d**).

Four consecutive cycles of the shape-memory test were carried out for all aliphatic PBIs (Figure 5a–c), and the results are summarized in Figure 5d. In all cases, the R_f_ values are >99%, indicating that the temporary shapes of aliphatic PBIs could almost be completely fixed, which is mainly explained by the significant difference in E_g_’ and E_r_’.

The R_r_ values from the first testing cycle are 53.9%, 83.8% and 78.4%, respectively, for C7-PBI, C10-PBI and C14-PBI. However, during subsequent cycles of the shape-memory test, R_r_ substantially increases, reaching 91.8%, 95.6% and 93.3%, respectively, for C7-PBI, C10-PBI and C14-PBI. Generally, the difference in R_r_ values for the first and following shape-memory cycles is ascribed to inner stresses resulting from the processing history of the sample [43]. After the first shape-memory cycle, the materials’ properties become more homogeneous, which results in an improvement in the shape-recovery process. On the other hand, in benzimidazole-containing polymers, consecutive increases in R_r_ values could be explained by the formation of an ordered structure during shape-memory cycles [26,27].

**Figure 6:**
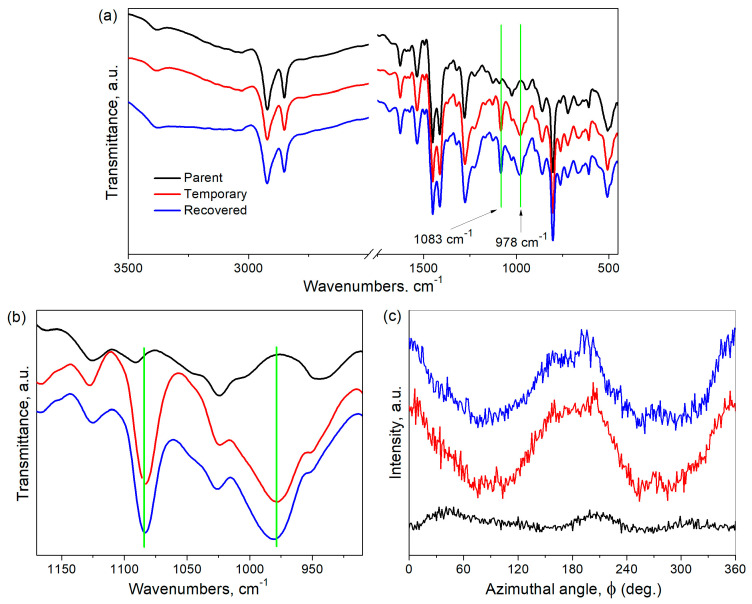
FTIR spectra (**a**,**b**) and intensity traces of the reflections around the azimuth (**c**) of the parent, temporarily shaped and recovered C10-PBI.

For the investigation of structural changes that occur during the shape-memory test, the parent polymers, as well as the temporarily shaped and recovered samples, were studied by FTIR spectroscopy (Figure 6a,b and Figure S1). In the spectra of the temporarily shaped and recovered samples compared to those of the initial polymers, the appearance of new peaks at ~1083 cm^−1^ and ~978 cm^−1^ is observed. Absorption bands in this region are usually ascribed to the skeletal vibrations of aliphatic groups [26]. Since these two peaks appear in the spectra of films with temporary shapes and remain unchanged for recovered samples, it could be concluded that after film stretching, the macromolecules adopt a thermodynamically favorable conformation and retain it after shape recovery. It should be noted that such phenomena are observed for all studied PBIs.

Conformational changes may be accompanied by the orientation of the macromolecules. For this reason, the parent polymers, temporarily shaped samples and recovered samples were investigated by WAXD azimuthal integration at 2θ = 15° − 25°, corresponding to the amorphous halo. The results (Figure 6c) show that the azimuthal intensity trace of the parent C10-PBI is flat. However, two peaks at 0° (360°) and 180° (parallel to the stretching direction) appear in the case of the temporarily shaped sample. Moreover, the azimuthal intensity keeps remains for the recovered sample. We note that C7-PBI and C14-PBIs demonstrate similar results; therefore, we do not display them. These data clearly prove the appearance of oriented structures during the shape-memory cycle. It should be noted that similar results on the formation of ordered structures in benzimidazole-based (co)polymers were previously reported [26,27].

Thus, based on the presented results, we can conclude that a thermodynamically favorable ordered structure is formed during the shape-memory test of aliphatic PBIs, and this results in a significant increase in R_r_ values.

Despite the fact that all PBIs show the same structural changes during the shape-memory test, the obtained values of R_r_ significantly differ. Since C7-PBI chains have the fewest short aliphatic groups (seven methylene units), it was expected that C7-PBI would demonstrate better recovery ability due to a higher ratio of hard and soft segments. However, as mentioned above, in the case of C7-PBI, a low R_r_ value of 91.8% is obtained. We assume that this fact is attributed to the lower molecular weight of C7-PBI in comparison to C10-PBI and C14-PBI. X. Xiao et al. reported that the decrease in the molecular weight of thermoplastic shape-memory polyimides results in a simultaneous decrease in R_r_ [43,46]. Moreover, similar results were obtained in the case of thermoplastic shape-memory polyurethanes [47]. It seems that, in the case of C10-PBI, an optimal balance between the molecular weight and the ratio of hard and soft segments is achieved, and thus, it demonstrates a high R_r_ (95.6%). A further increase in the number of methylene units (in the case of C14-PBI) results in a slight decrease in R_r_ to 93.3%, which could be explained by the reduced ratio of hard and soft segments.

Thus, the presented data clearly demonstrate that C10-PBI has an excellent shape-memory performance. Additionally, T_rec_ in this case reaches 258 °C, making it suitable for applications in high-temperature shape-memory fields.

**Figure 7:**
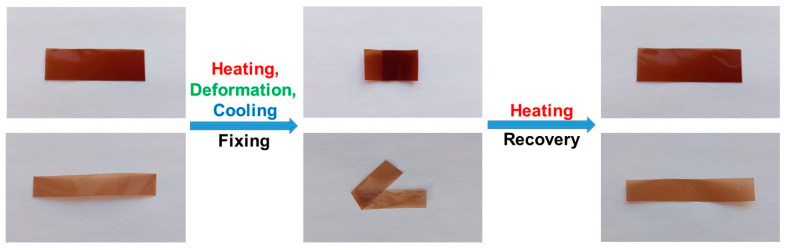
The shape-memory process of C10-PBI (**top**) and C14-PBI (**bottom**).

The shape-memory process of C10-PBI and C14-PBI is demonstrated in Figure 7. As can be seen, macroscopically, C10-PBI and C14-PBI can almost completely return to their original shapes after several seconds of heating at a temperature of T_g_ + 30 °C.

## 4. Conclusions

In summary, a series of new high-temperature shape-memory PBIs with flexible aliphatic segments were prepared by the polycondensation of DAB and aliphatic dicarboxylic acids in ER. It has been found that the length of the methylene chain in dicarboxylic acid significantly influences the molecular weight of the resulting polymers and, as a result, their mechanical properties and shape-memory performance. C10-PBI based on DAB and dodecanedioic acid possesses good shape-memory performance (R_f_ is 99.6% and R_r_ is 95.6% at T_rec_ = 258 °C) due to an optimal combination of high molecular weight and the ratio of hard benzimidazole and soft aliphatic segments. We note that the shape-memory step has to be repeated after the first cycle to achieve high values of R_r_, and the improvement of the shape-memory process is due to the formation of ordered structures involving hydrogen bonds between benzimidazole rings. Due to high mechanical strength (129.3 ± 7.1 MPa), thermal stability (T_10%_ = 464 °C) and good shape-memory performance, C10-PBI shows promise for practical use in advanced technologies, including the aerospace industry.

## Figures and Tables

**Table 1:** Conditions of PBIs’ synthesis.

Polymer	Dicarboxylic Acid Used	Reaction Conditions			η_red_ *, dL/g	Film-Forming Ability
		Temperature, °C	Duration, h	Monomer Concentration, mol/L		
C4-PBI	HOOC–(CH_2_)_4_–COOH adipic acid	120	10	0.2	0.72	No
C7-PBI	HOOC–(CH_2_)_7_–COOH azelaic acid		5	0.2	6.17	Yes
C10-PBI	HOOC–(CH_2_)_10_–COOH dodecanedioic acid		5	0.2	8.39	Yes
C14-PBI	HOOC–(CH_2_)_14_–COOH hexadecanedioic acid		1.5	0.13	10.03	Yes

* Measured in concentrated FA.

**Table 2:** Thermal and mechanical properties of PBIs.

Sample	TGA		Mechanical Test	
	T_10%_, °C ^1^	Char Yield, % ^2^	Tensile Strength, MPa	Elongation at Break, %
C7-PBI	470	17	95.5 ± 6.2	19.8 ± 1.9
C10-PBI	464	23	129.3 ± 7.1	32.3 ± 2.6
C14-PBI	460	17	83.9 ± 5.3	39.1 ± 3.1

^1^ The temperature at which 10% weight loss occurs; ^2^ residual weight percentage at 800 °C in argon.

## Data Availability

The data presented in this study are available on request from the corresponding author.

## Supplementary Materials

The following supporting information can be downloaded at https://www.mdpi.com/article/10.3390/polym15061399/s1. Figure S1: FTIR spectra of parent C7-PBI (a) and C14-PBI (b) films with temporary shapes and recovered samples after the first shape-memory cycle; Table S1: Solubility of PBIs.
