# Supplementary material for: Aliphatic Polybenzimidazoles: Synthesis, Characterization and High-Temperature Shape-Memory Performance
Source: Polymers (Basel). 2023 Mar 11;15(6):1399. doi: 10.3390/polym15061399 (PMC10055794; doi:10.3390/polym15061399)
Supplement: Supplementary file 1 [file polymers-15-01399-s001.zip › polymers-2204463-supplementary.pdf]

Supplementary data

Aliphatic Polybenzimidazoles: Synthesis, Characterization and High Temperature Shape Memory Performance

Bato Ch. Kholkhoev[1*], Zakhar A. Matveev[1], Kseniia N. Bardakova[2,3], Peter S. Timashev[2,3,4,5], Vitaliy F. Burdukovskii[1]

[1]Baikal Institute of Nature Management, Siberian Branch of the Russian Academy of Sciences, Sakhyanovoy str. 6, 670047 Ulan-Ude, Russia

[2]Institute of Photonic Technologies, Research center “Crystallography and Photonics”, Russian Academy of Sciences, Pionerskaya str. 2, 108840 Troitsk, Moscow, Russia

[3]Institute for Regenerative Medicine, Sechenov University, Trubetskaya str. 8-2, 119991 Moscow, Russia

[4]Semenov Institute of Chemical Physics, Russian Academy of Sciences, Kosygina str. 4, 119991 Moscow, Russia

[5]Lomonosov Moscow State University, Chemical Department, Leninskiye Gory 1-3, 119991 Moscow, Russia

[*]Corresponding author, e-mail: holh_bat@mail.ru

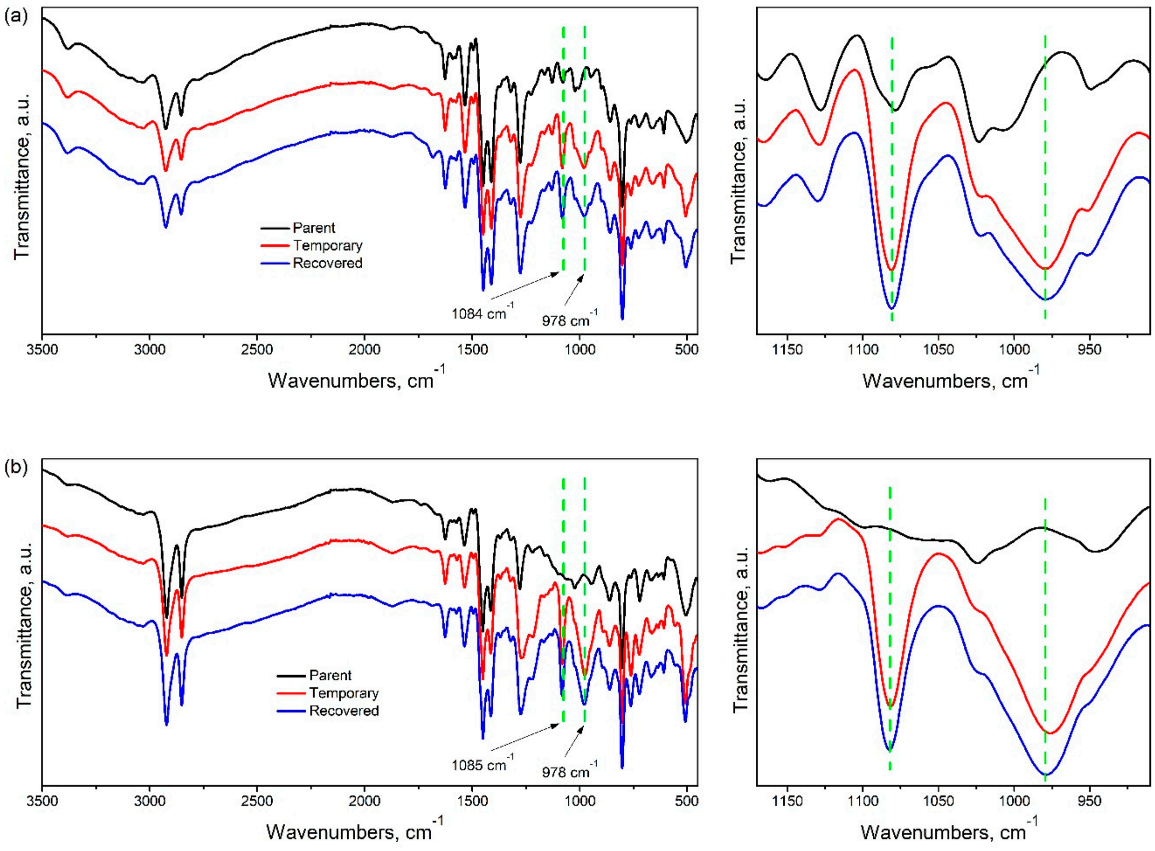

Figure S1. FTIR spectra of parent C7-PBI (a) and C14-PBI (b), films at a temporary shape and recovered samples after the first shape memory cycle.

Table S1. Solubility of PBIs.

Sample	H_2SO_4	HCOOH	DMSO	NMP	DMF	DMAc
C7-PBI	++	++	+–	–	–	–
C10-PBI	++	++	+–	–	–	–
C14-PBI	++	++	–	–	–	–

++: Soluble at ambient temperature. +–: partially soluble after heating.

–: insoluble even after heating.
